# Potential Predictors and Prevalence of Helicobacter pylori Infection Among Adult Patients With Dyspepsia: A Retrospective Study From Qatar

**DOI:** 10.7759/cureus.16216

**Published:** 2021-07-06

**Authors:** Vamanjore A Naushad, Nishan K Purayil, Ahmad Badi, Prem Chandra, Hassan O Abuzaid, Mohamed Milad Abuhmaira, Abdo Lutf, Firjeeth Paramba, Irfan Varikkodan, Abdel-Naser Y Elzouki

**Affiliations:** 1 Internal Medicine, Hamad Medical Corporation, Doha, QAT; 2 Clinical Department, College of Medicine, Qatar University, Doha, QAT; 3 Clinical Medicine, Weill Cornell Medicine - Qatar, Doha, QAT; 4 Medical Research Center, Hamad Medical Corporation, Doha, QAT

**Keywords:** helicobacter pylori, h. pylori, clo test, dyspepsia, peptic ulcer disease, gastritis

## Abstract

Aim

To study the prevalence of Helicobacter pylori (H. pylori) infection among dyspeptic patients of various ethnic origins in Qatar and determine the association between H. pylori infection and various demographic factors and endoscopic findings.

Methods

A retrospective data review was carried at Alkhor Hospital, Hamad Medical Corporation, Qatar. Adult patients who underwent endoscopy for the evaluation of dyspepsia between January 2011 to December 2017 were included. Patients who underwent endoscopy for reasons other than dyspepsia and those with incomplete data were excluded.

Results

Of the 638 subjects included, 58.9% were males, and the mean age of the subjects was 42.2 years (range 18-79 years). Epigastric pain (80.6%) was the most common symptom, followed by heartburn (26.2%). Forty point nine percent (40.9%) had a positive Campylobacter-like organism (CLO) test for H. pylori. A higher prevalence of H. pylori infection was observed among subjects between 31-50 years of age (43.6%) and 18-30 years (40.5%), and in Asian (42.2%) and Middle East and North African nationals (MENA) nationals (40%). Among the endoscopic findings, esophagitis (P=0.002) and gastritis (P=0.001) showed a statistically significant correlation with H. pylori positivity. Univariate regression analysis revealed an increased risk for H. pylori infection among all age groups except above 65 years, with an odds ratio (OR) of more than 2 in all the three age groups. Among various ethnicities, patients from Asia and MENA countries showed an increased risk of getting H. pylori infection (OR 1.16, 95% CI; 0.77,1.75 and OR 1.06, 95% CI 0.70,-1.61 respectively). The multivariable logistic regression analysis showed that subjects with endoscopic findings of esophagitis (adjusted OR 1.67, 95%CI 1.19, 2.34; P=0.003), gastritis (adjusted OR 1.79, 95%CI 1.27, 2.57; P=0.001), and duodenal ulcer (adjusted OR 2.41, 95%CI 1.24, 4.70; P=0.010) remained significantly associated with an increased risk of having H. pylori infection.

Conclusion

The burden of H. pylori infection in patients with dyspepsia undergoing endoscopy is not low in Qatar. Less than 65 years of age, Asian nationals, and being from the MENA region were the demographic predictors for H. pylori infection. The finding of esophagitis, gastritis, and duodenal ulcer on endoscopy were independent endoscopic predictors for having H. pylori infection.

## Introduction

Helicobacter pylori (H. pylori) is a gram-negative, spiral-shaped bacteria that colonizes gastric mucosa, which is thought to be acquired in childhood. Infection with H. pylori has been associated with peptic ulcer disease, chronic gastritis, and gastric malignancy [1-2]. In addition, H. pylori infection has also been reported in extra-digestive diseases such as hepatobiliary disease [3-4], idiopathic thrombocytopenic purpura (ITP) [5], ischemic heart disease (IHD) [6], and autoimmune thyroid disease [7]. The prevalence of H. pylori infection is 80%-90% in developing countries, whereas, in developed nations, the prevalence is much lower, 20%-50% [8]. The prevalence rates in the western world decreased significantly in the last two decades. A meta-analysis by James et al. reported that H. pylori prevalence rates in Europe after 2000 decreased from 48.8% to 39.8%, whereas, in northern America, it decreased from 42.7% to 26.6%. In the Oceania region, the rates decreased from 26.6% to 18.7% after 2000. However, the prevalence rates were nearly similar before and after 2000 in Asia (53.6 vs. 54.3%) and Latin America (62.8% vs 60.2%) [9]. The possible causes for higher prevalence include low socioeconomic status and living in crowded households [10-12]. The transmission mode includes ingesting contaminated food or water or direct contact between humans [13-16]. In addition, iatrogenic transmission during dental procedures and endoscopies have been reported [17].

The data regarding H. pylori infection in Qatar is limited with one study reporting a prevalence of 77% [18]. Qatar has a large expatriate population and not many studies have been done on the prevalence of H. pylori infection among various nationalities. Hence, we decided to compare H. pylori infection among patients from various ethnicities and age groups.

We aimed to evaluate the prevalence of H. pylori infection in patients with dyspepsia who underwent an endoscopy and study the association of H. pylori infection with various demographic factors and endoscopic findings.

## Materials and methods

Study design and setting

A retrospective study was carried out at Alkhor Hospital, Hamad Medical Corporation, Qatar.

Study subjects

Patients above the age of 18 who underwent an endoscopy to evaluate dyspepsia between January 2011 and December 2017 were included. Patients with incomplete data and those who underwent endoscopy for reasons other than dyspepsia were excluded from the study. The first endoscopy was taken as index one for patients who had more than one endoscopy during the study period. Dyspepsia was defined according to National Institute for Health and Care Excellence (NICE) guidelines, which describes dyspepsia as a range of symptoms that include upper abdominal pain or discomfort, heartburn, gastric reflux, nausea, or vomiting [19].

H. pylori diagnosis

H. pylori was diagnosed by the Campylobacter-like organism (CLO) test done on the biopsy specimen. The CLO test is a commercially available gel-based rapid urease test. The presence of H. pylori urease enzyme in the gastric biopsy specimen converts the urea test reagent to ammonia, which increases the pH, leading to color change in the pH monitor. These tests have 95%-100% specificity and sensitivity of 85%-95% [20]. 

Data collection

Data were retrieved from the medical records file and electronic database of patients using the health care number. Details about demographics, symptoms, smoking habits, alcohol consumption, nonsteroidal anti-inflammatory drugs (NSAID) use, endoscopic findings, and the results of the CLO test for H. pylori were noted. For analytical purposes, we grouped the study subjects into five groups based on ethnicity: Qatari nationals, Middle East and North African (MENA) nationals, Asians (excluding Qatar and other nations included in the MENA group), Africans (excluding the nations included in the MENA group ), and others.

Statistical analysis

Descriptive statistics were used to summarize and determine the sample characteristics and distribution of participants’ data. The normally distributed data and results were reported with mean and standard deviation (SD); the remaining results were reported with median and interquartile range (IQR). Categorical data were summarized using frequencies and proportions. The primary outcome variable in this study is the prevalence of positive H. pylori among dyspeptic patients undergoing endoscopy, and it was estimated and presented along with a 95% confidence interval (CI). Associations between two or more qualitative data variables were assessed using the chi-square (χ2) test or Fisher Exact test as appropriate. Quantitative data between the two independent groups (positive H. pylori vs negative H. pylori) were analyzed using unpaired t or Mann Whitney U test as appropriate.

Univariate and multivariate logistic regression analysis (controlling and adjusted for potential predictors and confounders such as age, gender, ethnicities, comorbidities, presenting symptoms, and endoscopic findings) were applied to determine and assess the associations and predictive values of predictors and confounders stated above with a binary outcome variable risk of developing H. pylori infection. The results of logistic regression analyses were presented as odds ratio (OR) with corresponding 95% CI. The receiver operating characteristic curve (ROC) was computed and constructed to evaluate and assess the predictive accuracy and discriminative ability of the developed logistic regression model (based on the predicted probabilities) using potential significant variables found in the multivariate logistic regression model. All P values presented were two-tailed, and P values <0.05 were considered statistically significant. All statistical analyses were done using the statistical software packages SPSS version 27.0 (Armonk, NY: IBM Corp) and Epi-Info (Centers for Disease Control and Prevention, Atlanta, GA).

## Results

Demographic characteristics and symptoms

After exclusion, 638 files were reviewed for final analysis. The mean age of the patients was 42.2 years (range 18-79), and 376 (58.9%) were males. The majority were in the 31-50 years age group (55.3%). Most of the study group consisted of patients from Asia 218 (34.2%) and MENA countries (200; 31.3 %). There were 171 (26.8%) Qatari nationals. Epigastric pain (80.6%) was the most common symptom, followed by heartburn (26.2%) in the overall study subjects. The detailed basic demographic characteristics and symptoms are summarized in Table 1.

**Table 1:** Baseline demographics and clinical characteristics MENA: Middle East and North Africa; NSAID: nonsteroidal anti-inflammatory drug

Variables	Number (%) (N=638 )
Age group (in years)	
18-30	121 (19)
31-50	353 (55.3)
51-65	134 (21)
≥ 65	30 (4.7)
Gender	
Male	376 (58.9)
female	262 (41.1)
Nationality	
Qatar	171 (26.8)
MENA	200 (31.3)
Asia	218 (34.2)
Africa	36 (5.6 )
Others	13 (2.0)
Symptoms	
Epigastric pain	514 (80.6)
Belching	56 (8.8)
Nausea	65 (10.2)
Vomiting	61 (9.6)
Heart Burn	167 (26.2)
Early satiety	15 (2.4)
Melena	38(6 )
Co-Morbidities	
Hypertension	82 (12.9)
Diabetes Mellitus	76 (11.9)
Asthma	17 (2.7)
Coronary Artery Disease	16 (2.5)
Chronic Kidney Disease	5 (0.8)
Liver Disease	18 (2.8)
Medications	
NSAID	36 (5.6)
Steroid	3 (0.5)

Prevalence of H. pylori infection

Out of 638 patients, 40.9% (95% CI 37.2, 44.8) had a positive CLO test for H. pylori, and 59.1 (377) had a negative CLO test.

H. pylori prevalence by gender, age group, and ethnicity

There was no significant difference in H. pylori infection prevalence between males and females (41.2% vs. 40.5%, P=0.847). A higher prevalence of H. pylori infection was observed among subjects between 18-30 years (40.5 %, 95% CI 32.2, 49.4) ) and 31-50 years (43.6%, 95% CI 38.6, 48.8)) compared to other age groups. The rate of prevalence showed a decreasing trend in subjects above 50 years. However, the difference in prevalence among various age groups was not statistically significant (P=0.147). On subanalyzing the prevalence in various ethnicities, the prevalence was higher in Asian nationals (42.2%, 95% CI 35.8, 48.8) and MENA nationals (40%, 95% CI 33.5, 46.9, ). Among Qatari nationals, the prevalence was 38.6%. Even though the prevalence was found to be 50% in subjects from Africa, the total number of subjects in that group was significantly lower than in other groups. The difference in the prevalence of H.pylori infection among various ethnicities was statistically not significant (p .0678) (Table 2).

**Table 2:** Prevalence of H. pylori-positive by demographics and other clinical characteristics CI: confidence interval, N: total number, n: H. pylori-positive

Variables	n/N	Percent Prevalence (95% CI)	P-value
Overall	261/638	40.9 (37.2, 44.8)	
Gender			
Male	155/376	41.2 (36.4, 46.3)	0.847
Female	106/262	40.5 (34.7, 46.5)	
Age groups (years)			
18-30	49/121	40.5 (32.2, 49.4)	0.147
31-50	154/353	43.6 (38.6, 48.8)	
51-65	51/134	38.1 (30.3, 46.3)	
>65	7/30	23.3 (11.8, 40.9)	
Nationality			
Qatar	66/171	38.6 (31.6, 46.1)	0.678
MENA	80/200	40 (33.5, 46.9)	
Asia	92/218	42.2 (35.8, 48.8)	
Africa	18/36	50 (34.5, 65.5)	
Others	5/13	38.4 (17.7, 64.5)	
Symptoms			
Epigastric pain	211/514	41.1 (36.9, 45.4)	0.882
Belching	19/56	33.9 (22.9, 47.0)	0.266
Nausea	26/65	40 (29.1, 52.1)	0.875
Vomiting	22/61	36.1 (25.2, 48.6)	0.418
Heartburn	68/167	40.7 (33.6, 48.3)	0.954
Early satiety	6/15	40 (19.8, 64.3)	0.942
Melena	17/38	44.7 (30.2, 60.3)	0.621
Comorbidities			
Hypertension	30/82	36.6 (27.1, 47.4)	0.394
Diabetes mellitus	28/76	36.8 (26.9, 48.1)	0.442
Asthma	5/17	29.4 (13.3, 53.1)	0.328
Coronary artery disease	5/16	31.3 (14.2, 55.6)	0.426
Chronic kidney disease	0/5	0 (0, 43.5)	0.062
Liver disease	10/18	55.6 (33.7, 75.4)	0.200
Medications			
Steroid	0/3	0 (0, 56.2)	0.149
NSAID	10/36	27.8 (15.9, 44.1)	0.099

Correlation of H. pylori infection and endoscopic findings

In the included study subjects, 595 (93.3%) had an endoscopically identifiable cause of dyspepsia. Among 261 patients who were positive for H. pylori, only 12 (4.5%) had normal endoscopy, whereas, among 377 patients who were negative for H.pylori, 31(8.2%) had normal endoscopy.

In the overall study group, gastritis (67.2 %) was the most common endoscopic abnormality, followed by esophagitis (33.5%). Gastric and duodenal ulcer was seen in 3.9% and 6.3% subjects, respectively. Carcinoma of the stomach was seen only in three subjects.

Among various endoscopic findings, esophagitis (P= 0.002) and gastritis (P= 0.001) showed a statistically significant correlation with H. pylori positivity. Thirteen out of 25 (52%, P= 0.032) subjects with gastric ulcer and 24 out of 39 (61.5%, P= 0.017) subjects with duodenal ulcer had H. pylori infection. Among three patients who had gastric cancer, only one had H. pylori positivity. Of patients with normal endoscopy, 27.9% were positive for H. pylori (Table 3).

**Table 3:** Endoscopic findings in various groups PUD: peptic ulcer disease; GERD: gastroesophageal reflux disease

Endoscopic Findings	Overall Total N=638	H pylori-positive N=261	H pylori-negative 377	P-value
Normal endoscopy	43 (6.7)	12 (27.9)	31 (72.1)	
PUD	595 ( 93.3)	249 (41.8)	346 (58.2)	0.732
Esophagitis/GERD	214 (33.5)	106 (49.5)	108 (50.5)	0.002
Gastritis	429 (67.2)	195 (45.5)	234 (54.5)	0.001
Gastric ulcer	25 (3.9)	13 (52)	12 (48)	0.250
Gastric cancer	3 (0.5)	1 (33.3)	2 (63.7)	0.789
Erosive duodenitis	77 (12.1)	34 (44.2)	43 (55.8)	0.437
Duodenal ulcer	40 (6.3)	24 (60)	16 (40)	0.011

Association of H. pylori positivity with various predictors

A univariate regression analysis was done for the following variables: gender, age groups, ethnic groups, comorbid conditions, symptoms, and endoscopic findings.

Regression analysis revealed an increased risk for H.pylori infection among all age groups except above 65 years of age, with an odds ratio (OR) of more than 2 in all three age groups. Among various ethnicities, patients from Asian, African, and MENA countries showed a positive risk of having H. pylori infection; however, it was statistically not significant.

Univariate regression analysis of endoscopic findings revealed that the highest predictor for H. pylori infection was found to be duodenal ulcer (unadjusted OR 2.29 95%CI 1.19, 4.39, P=0.011) followed by gastritis (unadjusted OR 1.81 95%CI 1.28, 2.56, P=0.001) and esophagitis (unadjusted OR 1.70, 95%CI 1.22, 2.38, P=0.002), and all were statistically significant. Gastric ulcers on endoscopy were also seen as potential endoscopic predictors for H. pylori infection; however, it was statistically insignificant (P>0.05). Also, patients having an abnormal finding of PUD on endoscopy were shown to be at an increased risk of having H. pylori infection (unadjusted OR 1.86, 95%CI 0.94, 3.69, P=0.073) as shown in Table 4.

**Table 4:** Association of various predictors with H. pylori: logistic regression analysis * absence of each respective comorbidity; † absence of each respective symptom; ** abnormal; each respective endoscopy finding was considered as a reference group PUD: peptic ulcer disease; CAD: coronary artery disease; CI: confidence interval; MENA: Middle East and North Africa

Variable	H. pylori-positive, n (%)	Unadjusted odds ratio (OR)	95% CI for OR	P-value
Gender				
Male	155 (41.2)	1.0 (reference)		
Female	106 (40.5)	0.97	0.70, 1.33	0.847
Nationality				
Qatar	66 (38.6)	1.0 (reference)		0.693
MENA	80 (40)	1.06	0.70, 1.61	0.783
Asian	92 (42.2)	1.16	0.77, 1.75	0.472
African	18 (50)	1.59 1.591	0.77, 3.28	0.208
Others	5 (38.4)	0.68	0.17, 2.73	0.588
Age group (years)				
≥65	7 (23.7)	1.0 (reference)		
18-30	49 (40.5)	2.24	0.89, 5.62	0.087
31-50	154 (43.6)	2.54	1.06, 6.08	0.036
51-65	51 (38.6)	2.02	0.80, 5.04	0.132
Comorbidity*				
Hypertension	30 (36.6)	0.81	0.50, 1.31	0.394
Diabetes	28 (36.8)	0.82	0.50, 1.35	0.442
Bronchial asthma	5 (29.4)	0.59	0.21, 1.71	0.328
CAD	5 (31.3)	0.65	0.22, 1.89	0.426
Liver disease	10 (55.6)	1.84	0.72, 4.72	0.200
Symptoms†				
Epigastric pain	211 (41.1)	1.03	0.69, 1.54	0.882
Belching	19 (33.9)	0.72	0.42, 1.29	0.266
Nausea	26 (40)	0.96	0.57, 1.62	0.875
Vomiting	22 (36.1)	0.80	0.46, 1.38	0.418
Heartburn	68 (40.7)	0.99	0.69, 1.42	0.954
Early satiety	6 (40)	0.96	0.34, 2.74	0.942
Melena	17 (44.7)	1.18	0.61, 2.29	0.621
Endoscopy Findings**				
PUD	249 (41.8)	1.86	0.94, 3.69	0.073
Esophagitis	106 (49.5)	1.70	1.22, 2.38	0.002
Varices	8 (57.1)	1.96	0.67, 5.70	0.212
Hiatus hernia	2 (20)	0.36	0.08, 1.69	0.175
Gastritis	195 (45.5)	1.81	1.28, 2.56	0.001
Gastric ulcer	13 (52)	1.59	0.72, 3.55	0.250
Carcinoma stomach	1 (33.3)	0.72	0.66, 8.10	0.789
Duodenitis	34 (44.2)	1.16	0.72, 1.88	0.537
Duodenal ulcer	24 (60)	2.29	1.19, 4.39	0.011

**Figure 1:**
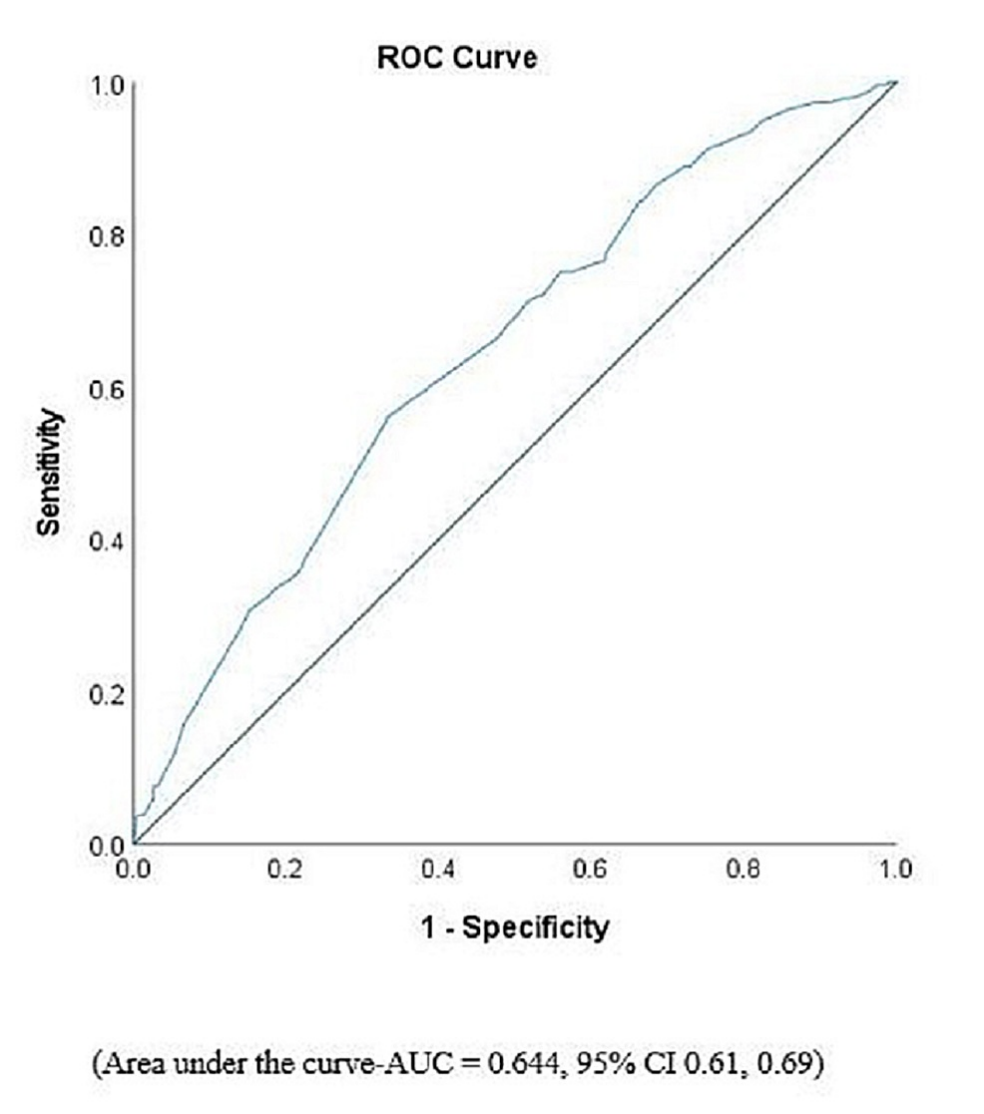
Receiver operating characteristic curve (ROC) to evaluate and assess the predictive accuracy of the developed logistic regression model (using the predicted probabilities)

The multivariable logistic regression analysis showed that subjects with endoscopic findings of esophagitis (adjusted OR 1.67, 95%CI 1.19, 2.34; P=0.003), gastritis (adjusted OR 1.79, 95%CI 1.27, 2.57; P=0.001), and duodenal ulcer (adjusted OR 2.41, 95%CI 1.24, 4.70; P=0.010) remained significantly associated with increased risk of having H. pylori infection after controlling and adjusting for all other potential confounder and predictors such as age, gender, ethnicities, comorbidities, presenting symptoms and other endoscopic findings shown in Table 4. Thereafter, we computed a prediction model using ROC analysis to evaluate the discriminative ability of potentially significant variables that indicated and demonstrated a modest fit (area under the curve (AUC)=0.644, 95% CI 0.61, 0.69) using potential predictors and risk factors observed in the developed multivariate logistic model as shown in Figure 1 and Table 5.

**Table 5:** Association of various predictors with H. pylori: multivariate logistic regression analysis * abnormal; each respective endoscopy finding was considered a reference group.

Variable	H. pylori-positive, n (%)	Adjusted odds ratio (OR)	95% CI for OR	P-value
Endoscopy Findings*				
Esophagitis	106 (49.5)	1.67	1.19, 2.34	0.003
Gastritis	195 (45.5)	1.79	1.27, 2.57	0.001
Duodenal Ulcer	24 (60)	2.41	1.24, 4.70	0.010

## Discussion

The results showed that the overall prevalence of H. pylori infection was 40.9% in the study subjects, which is lower than that reported from Qatar in the past. Latif et al. studied the H. pylori infection in patients who underwent esophageal gastroduodenoscopy and reported that 77% of their study subjects were positive [18]. The higher prevalence in their study could be because they diagnosed H. pylori infection by histopathology examination. In contrast, supporting our results, studies from other regions of the Middle East reported lower prevalence. Published reports from Saudi Arabia reported a varying range of H. pylori infection prevalence in dyspeptic patients. Mohammad Akeel et al. [21] reported a prevalence of 46.5%, whereas Ayoob et al. [22] found it to be 54.9%. Higher prevalence rate than our results were reported from Kenya (71%) [23], Nigeria (64%) [24], Pakistan (57%) [25], Cameron [26], and Turkey (65.9%) [9].

Studies from the western world reported a wide varying range of prevalence. Lower prevalence rates are seen in countries like Switzerland (18.9%), Sweden (26.2%), and Denmark (22.1%). When countries like the United Kingdom (35.5%), Netherlands (35.5%), and Germany (35.3%) reported a moderate level of prevalence rates, Eastern European nations Poland (66.6%), Romania (68.5%), and the Russian Federation (78.5%) reported a higher level of prevalence [9]. This wide range of differences in prevalence rates in these countries could be due to the difference in economic status and life habits.

We also examined the prevalence of H. pylori infection in patients of various ethnicities. Our results showed that the prevalence among Qatari nationals was 38.6%. Among expatriate populations, patients from Asia and MENA nations had a higher prevalence than Qatari citizens (42.2% and 40%, respectively). A meta-analysis showed that central Asia had the highest prevalence rate (79.5%) and southeast Asia showing the lowest prevalence (43.1%). When Asian subcontinent countries, India( 63.5%), Nepal ( 70.1%), and Pakistan ( 81.0%) reported higher prevalence rates, Southeast Asian nations, Malaysia ( 28.6%) and Singapore ( 40.8%) showed a lower prevalence rate [9]. The majority of Asian subjects in the present study were from India, Pakistan, Nepal, and Bangladesh, and hence, it is not surprising that the Asian cohort in our study had higher prevalence rates. Even though a higher prevalence was seen among the subjects from Africa, the number of subjects from these countries was much smaller than other groups. This is the first study from Qatar that compared H. pylori prevalence among various ethnic groups and its predictors. The significance of this comparison gains importance in view of that the state of Qatar is home to a large expatriate population from all over the world. A study from Kuwait that compared the prevalence among Kuwaiti nationals and expatriates reported an overall prevalence of 49.7% H. pylori infection in dyspeptic patients, and it was significantly higher in expatriates than Kuwaitis (42.6% vs. 57.6% P= 0.004) [27]. However, the main difference from our study was that they included the expatriate population as a single cohort.

On analyzing the prevalence of H. pylori infection among various age groups, it was found that the prevalence was higher in subjects between 18 and 50 years of age (40.5% in 18-30 years and 43.6 % in 31-50 years). This is much higher than that reported by Corojan et al. who reported it to be around 11% in 18-29 years and 20%-24% in those aged 30-59 years ago [28].

We studied the possible predictors for H pylori infection using various variables. Our results showed that gender has no significant influence on H. pylori infection, whereas age below 65 years has a positive association with H. pylori infection. In contrast, a meta-analysis by Ana Ferro reported that men had a significantly higher odds ratio and prevalence ratio of H. pylori infection compared to women [29]. Past published studies have shown a significant association between age and H. pylori infection [30-31].

The univariate analysis also revealed a positive association for H. pylori infection with ethnicity. Patients from Asia and the MENA region had a positive association for H. pylori infection. Even though our results showed African ethnicity as a predictor for H. pylori infection, this can not be taken into consideration, as their sample size was small.

Multivariate analysis was done for predictors, which showed a significant positive association in univariate regression analysis, and it revealed that esophagitis, gastritis, and duodenal ulcer on endoscopy as independent predictors for H. pylori infection.

The present study has some limitations. First, since H. pylori were diagnosed by the CLO test, which is less sensitive than biopsy, this might have excluded some patients with H. pylori infection. Second, subjects from the western world and African nations (excluding MENA countries) in the study population were low, and hence the association of predictors for H. pylori infection in these ethnic groups was not possible. Lastly, gastritis was diagnosed based on endoscopic appearance and not by histopathology, which might have overestimated the diagnosis.

## Conclusions

In conclusion, the prevalence of H. pylori infection among adult patients with dyspepsia is not low in the Qatar population. The prevalence is higher in the 18-50 year age group and in subjects from Asia and the MENA region. Less than 65 years of age, Asian nationals, and being from the MENA region were common predictors for having H. pylori infection. On endoscopy, having a finding of PUD itself is found to be a predictor for H. pylori infection with esophagitis, gastritis, and duodenal ulcer having the highest risk.
